# Investigation of the curative effects of palm vitamin E tocotrienols on autoimmune arthritis disease *in vivo*

**DOI:** 10.1038/s41598-019-53424-7

**Published:** 2019-11-14

**Authors:** Zaida Zainal, Afiqah Abdul Rahim, Ammu Kutty Radhakrishnan, Sui Kiat Chang, Huzwah Khaza’ai

**Affiliations:** 1 Nutrition Unit, Department of Product Development and Advisory Services (PDAS), Malaysian Palm Oil Board, Bandar Baru Bangi, Selangor 43000 Malaysia; 2 Jeffrey Cheah School of Medicine and Health Sciences, Monash University Malaysia, Jalan Lagoon Selatan, 47500 Sunway, Selangor Malaysia; 3 South China Botanical Garden, Chiness Academy of Sciences, 510650 Guangzhou, Guangdong China; 4 Department of Biomedical Science, Faculty of Medicine and Health Sciences, Universiti Putra Malaysia, Serdang, 43400 Malaysia; 5 Key Laboratory of Plant Resources Conservation and Sustainable Utilization, Guangdong Provincial Key Laboratory of Applied Botany, South China Botanical Garden, Chinese Academy of Sciences, Guangzhou, 510650 China

**Keywords:** Medical research, Experimental models of disease, Rheumatic diseases

## Abstract

The tocotrienol-rich fraction (TRF) from palm oil contains vitamin E, which possesses potent antioxidant and anti-inflammatory activities. Rheumatoid arthritis (RA) is a chronic joint inflammatory disease characterised by severe joint pain, cartilage destruction, and bone erosion owing to the effects of various pro-inflammatory mediators and cytokines. Here, we investigated the therapeutic effects of TRF in a rat model of collagen-induced arthritis (CIA). Arthritis was induced by a single intradermal injection of collagen type II in Dark Agouti (DA) rats. Rats were then treated with or without TRF by oral gavage from day 28 after the first collagen injection. Arthritic rats supplemented with TRF showed decreased articular index scores, ankle circumferences, paw volumes, and radiographic scores when compared with untreated rats. The untreated arthritic rats showed higher plasma C-reactive protein levels (*p* < 0.05) and production of pro-inflammatory cytokines than arthritic rats fed TRF. Moreover, there was a marked reduction in the severity of histopathological changes observed in arthritic rats treated with TRF compared with that in untreated arthritic rats. Overall, the results show that TRF had beneficial effects in this rat model of RA.

## Introduction

Rheumatoid arthritis (RA) is a systemic autoimmune rheumatic disorder of unknown aetiology that affects mainly the joints^1^. In RA, joint inflammation is characterised by marked changes in the cartilage owing to the effects of pro-inflammatory mediators^2^, cartilage destruction^3^, infiltration of leukocytes^4^, and bone erosion^5^. Although the aetiology of RA is unclear, growing evidence suggests that most of the pathological features observed in the joints of patients with RA are related to the effects of various pro-inflammatory cytokines, such as tumour necrosis factor-α (TNF-α), interleukin (IL)-1α, and IL-1β^6^. These cytokines play key roles in modulating inflammatory responses in affected joints^7^. For example, TNF-α induces production of inflammatory mediators, such as cytokines (e.g., IL-1β and IL-1α) and matrix metalloproteinases (MMPs), which in turn cause cartilage and bone destruction^8^. Moreover, TNF-α also reduced synthesis of glycosaminoglycans. Some pro-inflammatory cytokines (e.g., TNF-α, IL-1α, and IL-1β) can cause increased expression of cyclooxygenase-2 (COX-2) in the inflamed tissues in arthritic joints^9^. COX-2 is an essential enzyme required for the production of pro-inflammatory eicosanoids, including prostaglandin E2. Hence, COX-2 is upregulated in many inflammatory conditions^10^. The orchestrated interactions of T and B cells together with pro-inflammatory cytokines play key roles in the pathophysiology of RA. Although the key cytokines directly implicated in this process are TNF-α and IL-6, IL-1β and IL-17 may also play important roles^11^. Hence, the development of biological agents that specifically target immune mediators may result in newer and more effective treatments for RA^12^. Many of the MMPs have been reported to play key roles in cartilage destruction, which is often observed in RA. These MMPs are generally produced by activated macrophages and fibroblasts in response to pro-inflammatory cytokines, such as IL-1β and TNF-α^13^.

Animal models have been used to investigate the pathogenesis of RA in order to elucidate the aetiology and pathophysiology of this disease^14^. The roles of cytokines, particularly IL-1 and TNF-α, in the pathogenesis of RA have been previously reported in a number of studies involving animal models of RA, which closely mimic human disease^15^. In addition, these experimental RA models have provided a substantial amount of information on the pathophysiology of this disease, which has helped facilitate the development of novel therapeutic strategies^16^.

Vitamin E is a naturally occurring antioxidant that has the ability to modulate immune responses. There are two major families of vitamin E, i.e., tocopherols and tocotrienols. Both families have four isomers (α, β, γ, and δ)^17^. Tocotrienol is reported to be a more powerful antioxidant and anti-inflammatory agent owing to its unsaturated side-chain and the presence of three carbon double bonds in its farnesyl isoprenoid tail compared with the saturated side chains of tocopherols^18^. The powerful antioxidant activity exhibited by palm tocotrienols has facilitated extensive laboratory-based and clinical research of human health^19^. Crude palm oil is rich in tocotrienols, containing up to 800 mg/kg α- and γ-tocotrienols, and the distributions of the different types of vitamin E in palm oil are approximately 30% tocopherols and 70% tocotrienols^20^. Vitamin E in palm oil is known as the tocotrienol-rich fraction (TRF). To date, many studies have supported the use of palm TRF as a therapeutic agent for the treatment of many clinical conditions, including asthma^20^ and cancer^21^. As a fat-soluble antioxidant and anti-inflammatory agent, vitamin E is strongly related to free radicals and oxidative stress. Indeed, vitamin E may mitigate the damage induced by free radicals by limiting the production of free radicals, thereby affecting many physiological functions, such as cytokine production^22^. Increased vitamin E uptake has been found to reduce oxidative stress, protect cartilage from inflammation, and prevent and/or delay chronic diseases. Many studies have shown that palm TRF may have applications as a nutritional supplement and natural adjuvant or alternative therapeutic agent, with no reported adverse effects^23–25^. Currently, treatment for RA relies heavily on the long-term use of nonsteroidal anti-inflammatory drugs (NSAIDS). However, the therapeutic effects of NSAIDS have been rather disappointing because these drugs exhibit various side effects and are unable to inhibit the progression of RA or reduce the severity of joint inflammation^26^. Hence, there is a need to search for an alternative therapeutic agent with minimal side effects.

Accordingly, in this study, we aimed to investigate the therapeutic effects of supplementation with palm TRF to reduce inflammation in the joints of rats with collagen-induced arthritis (CIA) and decrease the levels of pro-inflammatory mediators in the plasma.

## Results

### Effects of TRF supplementation on the body weights of arthritic rats

**Figure 1:**
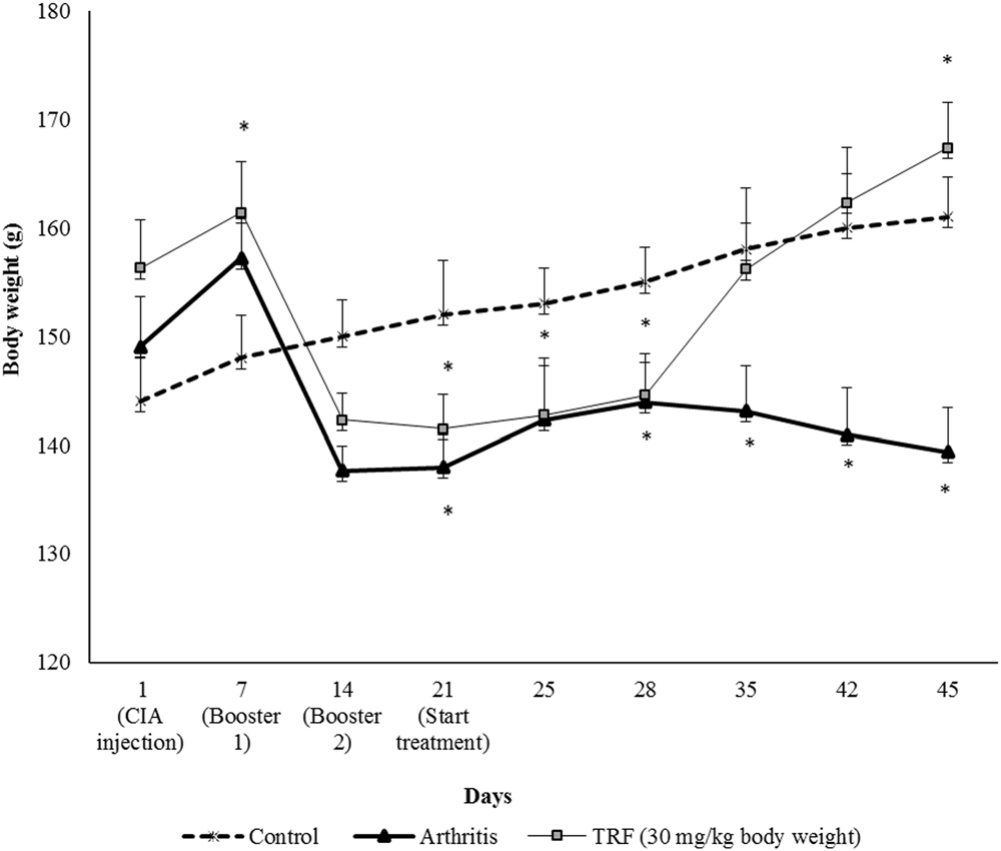
Effects of TRF on body weight in the (**a**) control (nonarthritic and without treatment), (**b**) arthritis (arthritis with vehicle), and (**c**) TRF (arthritis and supplemented with TRF [30 mg/kg body weight]) groups. Results were expressed as means ± standard deviations (n = 10 rats/group). *p < 0.05 compared with the arthritis group.

The body weight of each rat was recorded once per week from the start of the study (day 0) through day 45. The body weights of untreated Dark Agouti (DA) rats decreased sharply after the first booster injection on day 7. This downward trend continued after the second booster on day 14. In contrast, the body weights of arthritic rats supplemented with TRF increased steadily starting from day 21 (Fig. 1). These rats also had higher final body weights than untreated arthritic rats (*p* < 0.05).

### Effects of TRF on assessment of the severity of arthritis

**Figure 2:**
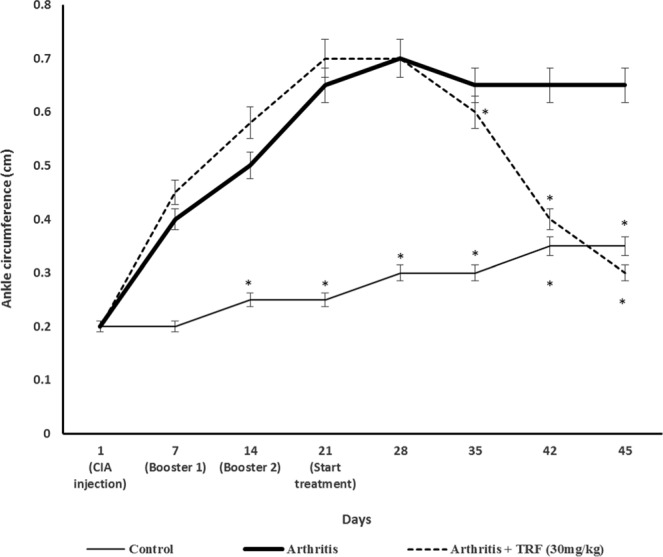
Ankle circumference in the (**a**) control (nonarthritic and without treatment), (**b**) arthritis (CIA with vehicle), and (**c**) arthritis + TRF (arthritis and supplemented with TRF [30 mg/kg body weight]) groups. Results are expressed as means ± standard deviations (n = 10 rats/group). *p < 0.05 compared with the arthritis group.

Paw oedema was evaluated by measuring paw diameter using digital callipers starting from day 0 until sacrifice, as described previously^27^. The rats began to show signs of inflammation in their joints on day 7 following induction of CIA. The inflammation progressed steadily from day 7 through day 28. Notably, none of the rats displayed any signs of arthritis, reduced mobility, or paw deformities before CIA induction, as demonstrated by the similar mean ankle circumferences (0.2 cm) in the rats from all groups before induction of CIA (Fig. 2). There was a marked (*p* < 0.05) increase in paw oedema in vehicle-treated arthritic rats (Fig. 2). However, arthritic rats treated with TRF displayed a significant (*p* < 0.05) reduction in paw oedema compared with that in vehicle-fed arthritic rats. The reduction was comparable to that observed in rats from the nonarthritic control group. Indeed, following TRF treatment, the mean ankle circumference in arthritic rats decreased sharply, reaching 0.2 cm (i.e., the same as that in the nonarthritic control) after 45 days. Notably, the mean ankle circumference in TRF-treated arthritic rats was significantly (*p* < 0.05) reduced from day 35 onwards.

**Figure 3:**
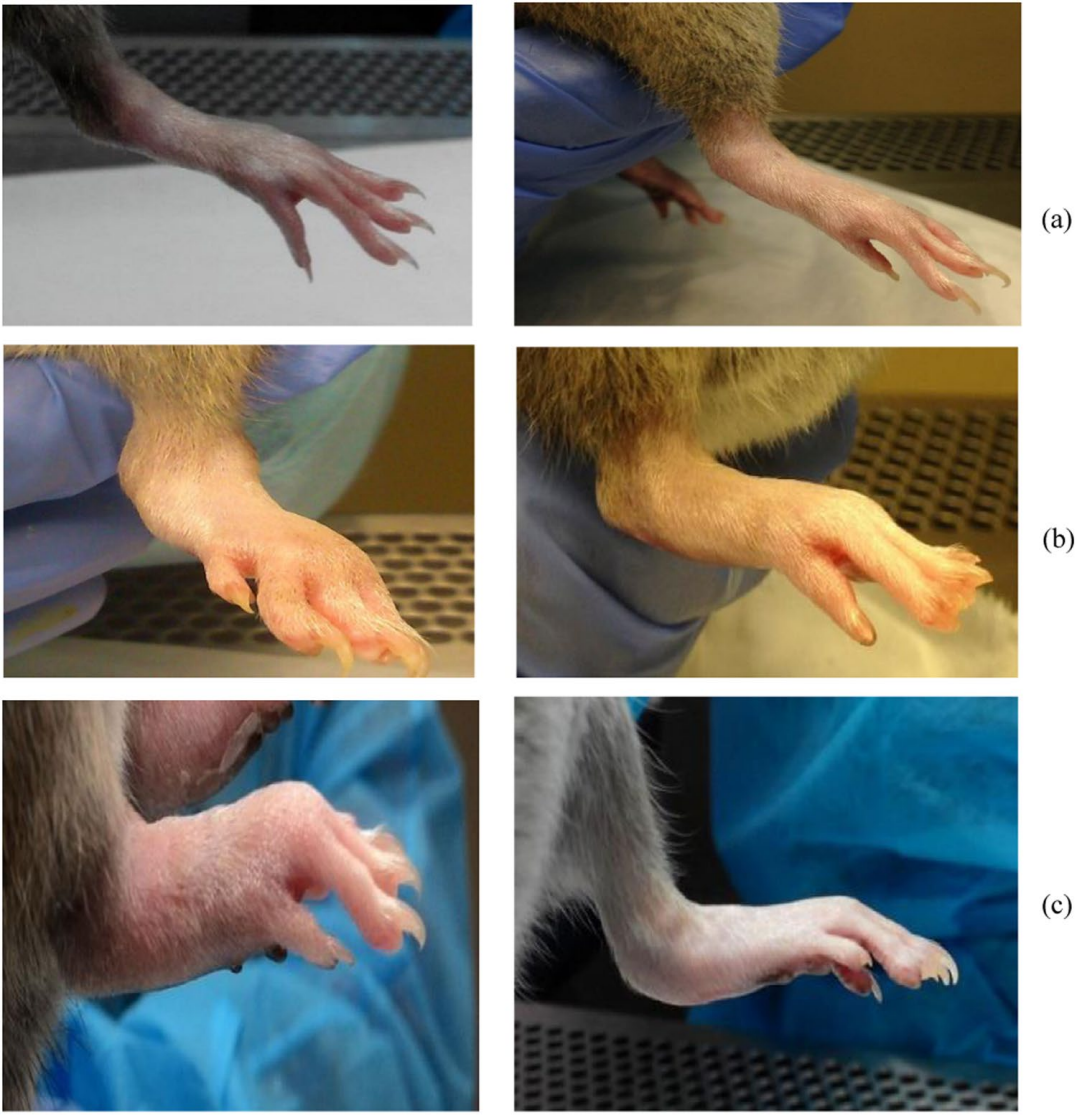
Gross morphology showing paw oedema in the hind paws of experimental animals on day 20 after CIA (left panel) and day 49 after treatment (right panel). (**a**) Control normal rats (without CIA and without treatment) showed no signs of joint inflammation. (**b**) Arthritic rats (arthritis only) showed maximal swelling and redness over joints. (**c**) Arthritic rats supplemented with TRF (30 mg/kg body weight) showed reduced severity of paw inflammation.

The gross morphologies of the hind paws of TRF-treated and untreated arthritic rats is shown in Fig. 3. The paws of untreated arthritic rats showed maximal swelling and redness over the joints (Fig. 3b). In contrast, a marked reduction in the swelling and redness of the joints was observed in TRF-treated arthritic rats (Fig. 3c). As treatment progressed, the mobility of the TRF-treated arthritic rats gradually improved. All TRF-treated arthritic rats recovered their capacity to move a few days after TRF treatment compared with that in untreated arthritic rats. Supplementation with TRF commenced on day 21, and the animals were fed daily until day 45.

### Effects of TRF on histological findings of arthritic paw joints

**Figure 4:**
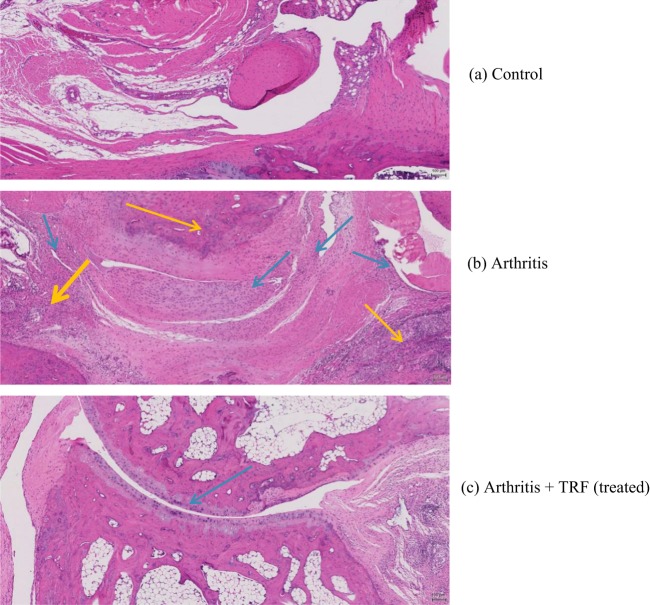
Photomicrographs of tarsal joints to compare histological changes in synovial inflammation in vehicle-fed and TRF-fed arthritic rats. (**a**) Joints of control rats (nonarthritic), showing a normal joint with no infiltrates in the synovium. (**b**) Joints of arthritic rats (fed vehicle), showing severe infiltration of inflammatory cells into the synovium. (**c**) Joints of arthritic rats treated with TRF, showing huge intact articular cartilage with mild inflammatory cell infiltration into the synovium (yellow arrows: peri-articular inflammation, blue arrows: synovial inflammation).

Haematoxylin and eosin (H&E)-stained sections from the hind paw joints of arthritic rats fed vehicle (untreated) or TRF were examined by light microscopy. Photomicrographs obtained from hind paw joints of arthritic and normal DA rats (no arthritis) were compared (Fig. 4). Histological analysis of the joints from normal DA rats (no arthritis) showed healthy joints with no signs of synovial hyperplasia, inflammatory cell infiltration, or tissue destruction (Fig. 4a). In contrast, joints from vehicle-fed arthritic rats showed marked synovial inflammation, fibrovascular pannus, cartilage degeneration/erosion, bone resorption, and peri-articular inflammation (Fig. 4b).

In arthritic rats, loss of articular cartilage and subchondral bone appeared to be accompanied by fibrous adhesions between the tarsal bones, as well as cartilage and bone remodelling. Histopathological findings of inflammation and degenerative changes in all tarsal joints were consistent with CIA^28^. Moreover, the histopathological changes observed in the synovium of vehicle-fed arthritic rats appeared to be quite severe when compared with that from TRF-treated arthritic rats. In vehicle-fed arthritic rats, severe erosion and degeneration of the cartilage were observed. In addition, the cartilage surface appeared rough with clumping of cartilage cells, and the joints showed extensive oedema with narrowing of the joint spaces. In contrast, in TRF-fed arthritic rats, the cartilage was smooth, and increased numbers of cartilage cells were observed when compared to vehicle-fed arthritic rats (Fig. 4c). Furthermore, joint inflammation was moderate, and bone resorption was reduced following oral supplementation with TRF.

### Effects of TRF on plasma levels of pro-inflammatory mediators

**Figure 5:**
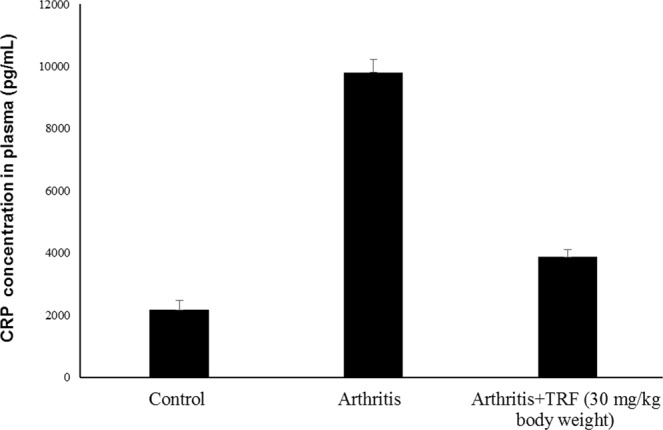
Effects of TRF on CRP levels in plasma. Serum samples were collected from the control, arthritis, and TRF-treated groups. The concentration of CRP was measured by enzyme-linked immunosorbent assay. Data are presented as means ± standard errors of the means (n = 10 rats/group). *p < 0.05 versus the control group.

**Figure 6:**
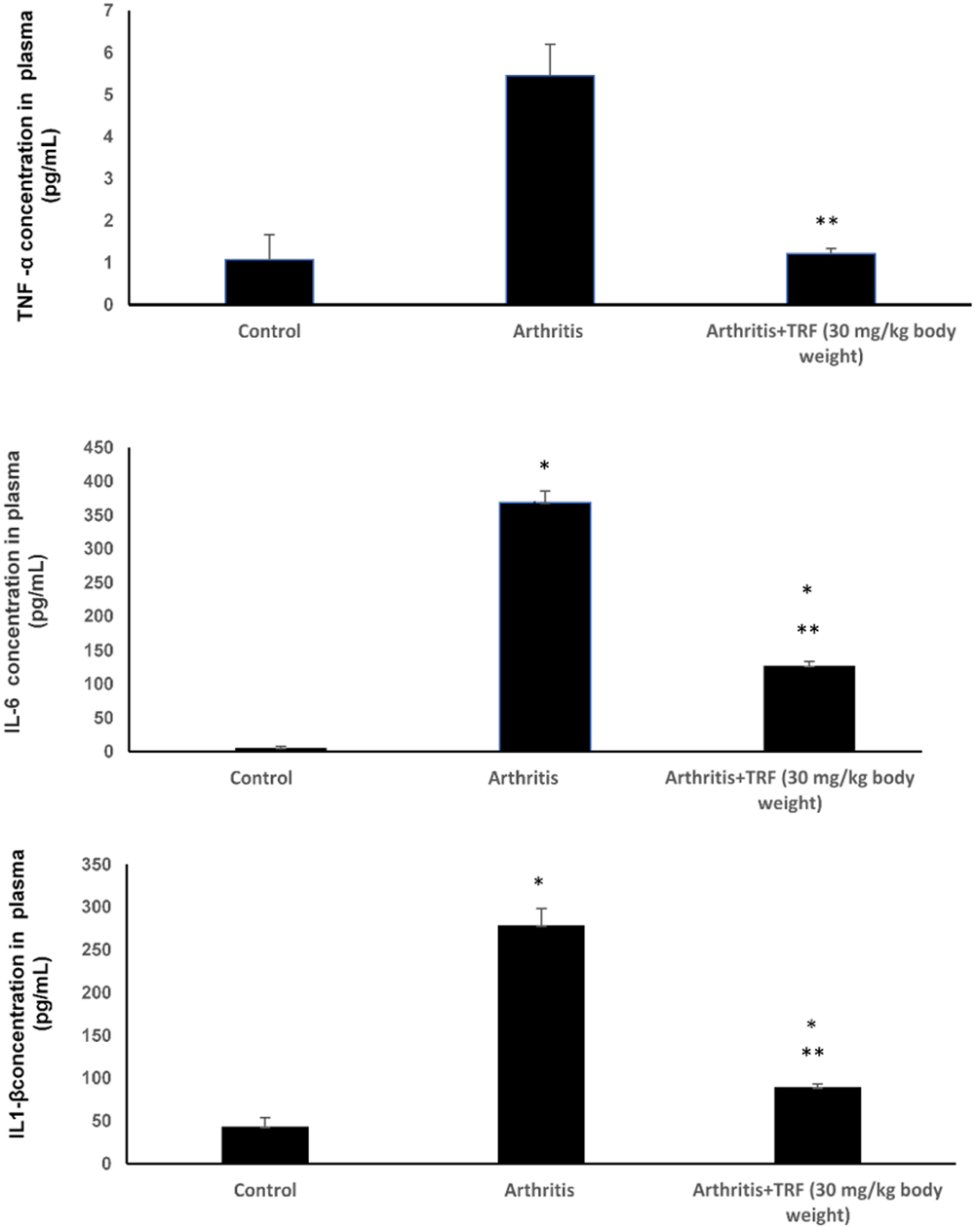
Plasma concentrations of (**a**) TNF-α, (**b**) IL-6, and (**c**) IL-1β measured before and after treatment using enzyme-linked immunosorbent assays. Data are presented as means ± standard errors of the means (n = 10 rats/group). *p < 0.05 compared with the control group, **p < 0.05 compared with the arthritis group.

Plasma levels of C-reactive protein (CRP) at the end of the study period are shown in Fig. 5. There was a marked (*p* < 0.05) increase in plasma CRP levels in vehicle-fed arthritic rats compared with that in control nonarthritic DA rats and TRF-fed arthritic rats. Plasma CRP levels in TRF-fed arthritic rats were comparable to those in control DA rats (Fig. 5). Similar findings were observed for plasma TNF-α (Fig. 6a), IL-1β (Fig. 6b), and IL-6 (Fig. 6c) levels. In all cases, there were marked (*p* < 0.05) increases in TNF-α, IL-1β, and IL-6 levels in the plasma from vehicle-fed arthritic rats compared with those in control nonarthritic DA rats and TRF-fed arthritic rats. The levels of TNF-α, IL-1β, and IL-6 in the plasma from TRF-fed arthritic rats were comparable to those in control nonarthritic DA rats.

### Analysis of bone mineral density (BMD) changes by computed tomography scanning

**Figure 7:**
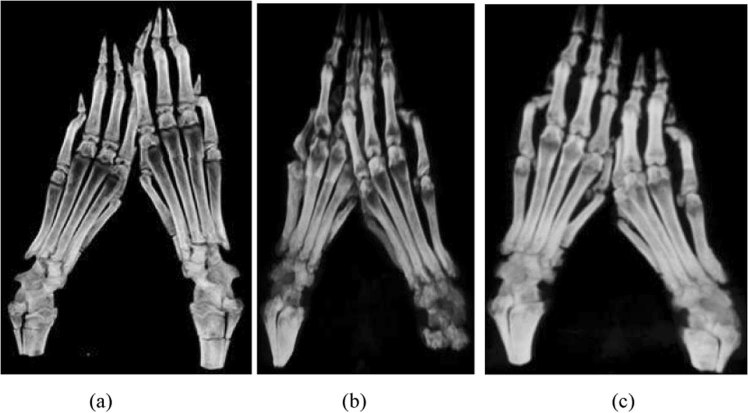
Effects of TRF on bone structure from (**a**) control rats (no arthritis and no treatment), showing a normal bone structure; (**b**) arthritic rats (vehicle-fed arthritis); and (**c**) arthritic rats fed TRF (30 mg/kg body weight).

To confirm the findings from serum biomarker analysis, we used computed tomography (CT) scanning to analyse changes in BMD in order to assess disease progression in CIA and to determine the disease index. In this analysis, the bone structures of arthritic rats from all experimental groups were subjected to scanning using a CT scanner 9 weeks after the first injection of collagen type II. The paws from vehicle-fed arthritic rats showed signs of erosive arthritis (Fig. 7b), as indicated by significant dark areas over almost all the joints (inflammation) when compared with paws from TRF-treated arthritic rats (Fig. 7b,c). Paws from TRF-treated arthritic rats showed reduced dark areas over the joints (Fig. 7c) when compared with arthritic paws from vehicle-fed arthritic rats (Fig. 7b). This observation suggested that TRF treatment reduced the level of bone destruction in CIA rats.

**Figure 8:**
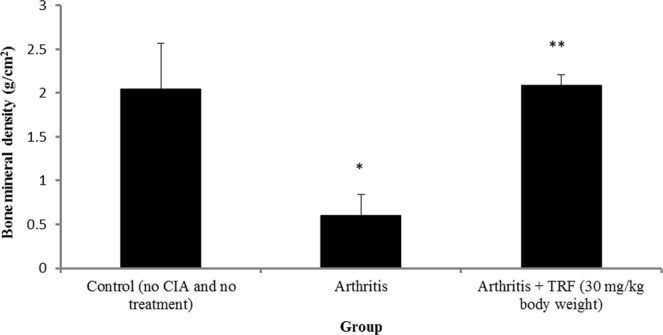
Changes in bone mineral density in the control group (no arthritis and no treatment), arthritis group, and arthritis group treated with TRF (30 mg/kg body weight). Data are presented as means ± standard errors of the means (n = 10 rats/group). *p < 0.05 compared with the control group, **p < 0.05 compared with the arthritis group.

The mean bone densities of rat bones were calculated from CT images (Fig. 8). The results from this analysis showed that the BMDs or thicknesses of each measured bone from vehicle-fed CIA rats were lower than those from control or TRF-fed CIA rats (Fig. 8). As shown in Fig. 8, the mean BMD in vehicle-fed CIA rats decreased compared with those in control and TRF-treated CIA groups. CIA rats with TRF treatment showed significant increases in BMD compared with that in untreated CIA rats (*p* < 0.05).

## Discussion

There are two main types of arthritis, i.e., RA and osteoarthritis (OA). RA affects patients of any age, causing swelling and pain in joints, and the symptoms differ from those of OA. In RA, immune cells attack the joints, causing severe inflammation, whereas in OA, natural wear and tear of joints due to control aging processes leads to arthritis^29^. The side effects of long-term consumption of NSAIDs limit the use of these agents in the treatment of RA; thus, there is an urgent need for the development of natural, safe, effective anti- inflammatory agents that can be used with minimal or no side effects.

As a powerful antioxidant and natural product, TRF from palm oil may be a promising therapeutic agent. TRF contains high amounts of γ- (30–39%), α- (14.6%), and δ-tocotrienols (8.0%) and a small amount of β-tocotrienol (3%) (Sime Darby, Malaysia). Several *in vitro* and *in vivo* studies have shown that the fraction of palm vitamin E and the tocotrienol fractions, which contain γ-tocotrienol and δ-tocotrienol, have anti-inflammatory activities, including the ability to inhibit inflammatory arthritis^30,31^. For example, Radhakrishnan *et al*.^30^ demonstrated the effects of palm γ-tocotrienol against oxidative stress and joint pathology in a rat model, whereas Haleagrahara *et al*.^31^ showed that palm δ-tocotrienol reduced inflammation in arthritic rats. Previous *in vitro* studies using calf chondrocyte cell cultures have demonstrated that γ-tocotrienol from palm oil can alleviate many of the discomforts of arthritic diseases^32^. Moreover, palm tocotrienol fractions, particularly γ-tocotrienol, downregulate several pro-inflammatory cytokines, e.g., TNF-α, IL-1α, IL-β, IL-6, and IL-8^30–32^. However, no reports have described the effects of palm TRF in alleviating joint destruction in the CIA rat model of RA.

The CIA rat model is reported to mimic human RA^33^; indeed, CIA rats show similar clinical signs, such as bone destruction, joint inflammation, and pro-inflammatory cytokine upregulation^34^. This model has been widely used for preclinical studies involving RA, including treatment and prognostic assessments. According to Kamada *et al*.^35^, DA rats are the most susceptible rat strain to develop arthritis when injected with type II collagen. Approximately 80–100% of collagen-treated animals will show joint pain beginning on days 21 and 28 after collagen injection. The sharp increase in joint pain observed in CIA rats is similar to that observed in patients with RA. In the current study, we examined the ability of TRF derived from palm oil to reduce inflammation in a rat model of RA.

In this animal model, collagen type II emulsified with Complete Freund’s adjuvant (CFA) was injected to female DA rats, which resulted in severe arthritis. The first sign of joint pain was observed on day 7, and the symptoms progressed to very severe arthritis by day 45; this condition can eventually develop into chronic arthritis. Arthritic rats showed restricted movement by approximately day 7. Indications of joint pain included swelling and redness over the joints, limited movement, and painfulness to the touch. Additionally, some rats developed a limp and moved around by dragging their paws. A slight decrease in body weight was observed due to changes in eating patterns. Arthritic rats fed TRF appeared to regain their mobility. These results were generally consistent with the findings of Radhakrishnan *et al*.^30^, who used γ-tocotrienol and δ-tocotrienol, and those of Haleagrahara *et al*.^31^ in CIA rat models.

In this study, TRF supplementation markedly reduced paw oedema. The paws of rats treated with TRF showed significantly reduced inflammation. Vehicle-fed CIA rats showed severe inflammation, but no changes in paw thickness. Thus, TRF treatment had considerable effects on reducing paw oedema. Hind paws were selected since they were influenced the most and have been shown to be critical in CIA. These findings suggested that TRF supplementation markedly reduced the severity of inflammation in the animals’ paws. Thus, TRF supplementation could alleviate some of the clinical symptoms of arthritis in this experimental rat model.

Body weight reductions are commonly observed as RA develops and are one of the most common symptoms observed in severe arthritis. The results from the current study on body weight changes were consistent with previous reports demonstrating that CIA significantly decreased body weight. In contrast, TRF feeding increased body weight compared with that in vehicle-fed CIA rats. The findings suggested that providing TRF supplementation to arthritic rats may have reduced their metabolism and blocked the generation of reactive oxygen species (ROS) in the tissues of arthritic rats, thus favouring fat accumulation.

CRP is an effective indicator of tissue damage or inflammation^36^ in the liver. CRP is formed in cartilage and bone tissues undergoing inflammation, as is observed in RA^37^. Increased plasma CRP levels are usually observed in response to inflammation in RA and other inflammatory diseases. Higher concentrations of CRP in the plasma indicate expanded joint changes in arthritis. Synovium of inflamed joints can trigger activation of macrophages and fibroblasts, inducing these cells to release more CRP^38^. Higher concentrations of CRP in the plasma is reported to indicate expanded joint changes in arthritis. In addition, inflammatory mediators such as, IL-1 and IL-6, are reported to be responsible for controlling inflammation and joint destruction in rheumatoid arthritis^39^. In the present study, CRP levels were significantly decreased following TRF supplementation, which could be due to inactivation of synovial macrophages and fibroblasts.

The results from this study showed that TRF supplementation reduced inflammation in arthritic joints, consistent with reduced plasma levels of CRP in these animals. Similar observations were reported by Radhakrishnan *et al*.^30^ and Haleagrahara *et al*.^31^ regarding the effects of γ-tocotrienol and δ-tocotrienol supplementation in CIA rats. However, the authors only used 5 mg/kg of the compound. Although this method is useful for determining the effectiveness of the treatments, evaluation of CRP levels is not sufficient for diagnosis of RA because this biochemical parameter is not specific for RA. Indeed, elevation of CRP levels can be indicative of various infections or inflammatory diseases^36^.

In severe arthritis, several pro-inflammatory cytokines are produced. These molecules are responsible for causing pain, redness, and painful swelling in the joints. Some of the most frequently reported inflammatory mediators include IL-1β and TNF-α^40^. In addition, activated CD4^+^ T helper cells present in the joints can activate other leukocytes to produce various other pro-inflammatory mediators and cytokines. For example, macrophages and synovial lining cells are stimulated to produce TNF-α, which plays a key role in inducing the production of cytokines, such as IL-1 and IL-6, in the synovial joints. TNF-α can act synergistically with IL-1β to induce production of other pro-inflammatory mediators, such as MMPs and prostaglandins^41^. Together, this milieu of pro-inflammatory mediators can damage the joints and cause joint pain. Higher TNF-α levels have been reported in patients or rats with arthritis^42^. Moreover, γ-tocotrienol^30^ and δ-tocotrienol^31^ supplementation can decrease joint swelling through mechanisms related to inhibition of inflammation. In the current study, we showed that TRF supplementation can represent an effective therapy by significantly reducing plasma levels of TNF-α levels in arthritic rats. The data showed that IL-1β and IL-6 (but not TNF-α) proteins were expressed in inflamed and treated joints. Notably, TRF treatment reduced IL-1β and IL-6 protein levels in accordance with reduced joint swelling.

Histological evaluation was performed in this study to determine the histological changes in disease severity between the groups, including synovial inflammation, cartilage degeneration, and bone erosion. Histological analysis was performed after the rats were euthanized on day 45. The histopathological and biomarker data were correlated with changes observed in paw oedema measurements. Some of the deformities observed in arthritic joints can result in functional deterioration and profound disabilities in the long term. The synovial membrane in CIA rats shows greater thickening of the synovial membrane than that in typical rats. The histopathological findings from the current study were consistent with those from previous studies, demonstrating extreme cartilage and bone destruction in addition to abundant infiltration of inflammatory cells in most of the joints examined from the CIA rats. However, in TRF-supplemented animals, histopathological analyses showed that TRF supplementation exerted strong anti-inflammatory effects, with marked reductions in vascularity, congestion, pannus growth, articular cartilage damage, subchondral bone damage, and decreased joint space.

Tocotrienols possess superior antioxidant and anti-lipid peroxidation effects compared with tocopherols. These superior activities of tocotrienols can be attributed to their ability to distribute within the membrane bilayer. Tocotrienols also have higher capacity to confine free radicals owing to the presence of unsaturated double bonds present in the tocotrienol structure^17^. TRF supplementation markedly reduced the progression of arthritis by blocking the pathogenesis of chronic inflammatory lesions and their progression to chronicity via improvements in cartilage destruction in RA. Future studies using a wider range of TRF doses and mechanistic studies are required to fully elucidate the effects of palm oil TRF on arthritis. In addition, human clinical trials should also be carried out to ascertain the effectiveness of TRF in patients with arthritis.

## Conclusion

In conclusion, our results showed that oral TRF supplementation attenuated the development of progressive joint destruction in rats with CIA. These findings suggested that treatment with TRF could reduce joint inflammation in the paws of CIA rats. Our observations showed that TRF exerted anti-arthritic effects by inhibiting the production of IL-1β, IL-6, TNF-α, and CRP. These inhibitory effects resulted in alleviation of arthritic symptoms in the experimental animals. These effects may have been partly related to the ability of palm oil TRF to reverse some of the histopathological changes caused by CIA and inhibit the production of pro-inflammatory cytokines, strongly suggesting that TRF may be a potential anti-arthritic agent. TRF treatment yielded promising outcomes (therapeutic effects) against CIA in this study. Based on the evidence from this study, TRF may be effective in reducing arthritis-related inflammation. However, our study had several limitations. First, we used a collagen-induced arthritis model, which did not completely mimic the pathogenesis of human RA. Second, the amount of TRF used in the animal may not be the same as that required for human patients with RA. However, investigating RA development in a preclinical model of arthritis in rats and the effects of TRF treatment provided key insights into the factors that could lead to disease and may facilitate the establishment of prevention strategies for RA. Further investigations are currently underway to evaluate TRF in the clinical setting in patients with RA.

## Materials and Methods

### Animals and materials

Female DA rats (4–5 weeks old) were purchased from Sterling Ascent Private, Ltd. (Australia). Collagen from chicken sternum (type II) and CFA were obtained from Sigma-Aldrich (St. Louis, MO, USA). TRF was obtained from Sime Darby Ptd. Ltd. (Selangor, Malaysia). Refined bleached deodorised (RBD)-stripped vitamin E oil was obtained from Malaysian Palm Oil Board-Engineering Department (Selangor, Malaysia). Acetic acid was obtained from Sigma-Aldrich. The digital callipers were from Senator Digital Callipers (Selangor, Malaysia). The CRP enzyme-linked immunosorbent assay (ELISA) kit was from Assay Pro (St. Charles, MI, USA). TNF-α, IL-1β, and IL-6 ELISA kits were purchased from NovateinBio (Novatein Biosciences, MA, USA). All other reagents and chemicals were purchased from Sigma-Aldrich.

### Animal care

Thirty female DA rats (4–5 weeks old) were used in this experiment. The rats were temporarily placed in a quarantine room for 2 weeks upon arrival at the Pre-Clinical Research Facility at the Malaysian Palm Oil Board (MPOB). The rats were then transferred to a specific pathogen-free room at the same facility. The rats were housed in individual ventilation cages and subjected to a 12-h light/12-h dark cycle. Food pellets (Altromin, Germany) and drinking water were provided *ad libitum*. All procedures involving the use of the experimental animals were performed in accordance with the National Animal Care Guidelines issued by the Institutional Animal Care and Use Committee of University Putra Malaysia (UPM). Ethical permission for the animal study was obtained from UPM in July 2014 (reference number UPM/IACUC/AUP-R042/2014).

### Arthritis induction and evaluation

After an acclimation period, the rats were injected with collagen that was emulsified in CFA. Briefly, 5 mg collagen from chicken sternum (type II) was dissolved in 2.5 mL cold acetic acid (0.1 M). This mixture was emulsified with 2.5 mL CFA and then homogenised for 15 min. This solution was prepared on ice to ensure that the protein in the emulsion was not denatured. The rats were anaesthetised, and arthritis was induced by injecting 0.05 mL collagen type II emulsified in CFA intradermally/subcutaneously into each paw of the hind limbs. Subsequently, two booster injections of the same concentration of ovalbumin were administered on day 7 (booster 1) and day 14 (booster 2), as shown in supplementary information, Supplementary Figure S1.

### Treatment with TRF

Rats were randomly divided into the control, arthritis, and arthritis-treated with TRF groups (n = 10 rats/group). The animals in the control group were fed 30 mg/kg body weight RBD stripped vitamin E (MPOB) daily and provided reverse osmosis water (Millipore Sigma, USA). The animals in the TRF-treated group received TRF (30 mg/kg body weight) daily through oral gavage starting from day 28 until the day of sacrifice. The TRF was prepared under Good Manufacturing Practice conditions. TRF contents (25% α-tocopherol and 75% tocotrienols) and purity were confirmed in a previous study^20^. The RBD stripped palm oil used as the vehicle was obtained from MPOB.

### Collection of blood plasma

Rats were sacrificed at the end of the study by CO_2_ asphyxiation. During necropsy, blood was withdrawn from all experimental animals through cardiac puncture. Blood samples (3–4 mL) were collected in vacutainer tubes containing ethylenediaminetetraacetic acid (EDTA). Plasma was isolated by centrifugation (4620 × *g* for 10 min at 4 °C; Eppendorf, Germany). The plasma samples were stored at −80 °C until further analyses.

### Quantification of pro-inflammatory mediators by ELISA

Plasma levels of CRP were assessed using a commercial kit purchased from AssayPro (St. Charles, MI, USA; Cat #ERC 1001-1) according to the manufacturer’s instructions. This kit was specific for measuring rodent CRP. Plasma levels of cytokines were determined using ELISA kits for TNF-α, IL-1β, and IL-6 (Novateinbio, Novatein Biosciences, MA, USA), according to the manufacturer’s instructions. The amounts of CRP, TNF-α, IL-1β, and IL-6 in the plasma were determined using a standard curve that was run for each of ELISA. Plasma levels of these pro-inflammatory mediators were quantified before CIA induction and at the end of the study.

### Histopathological studies

At necropsy, the fore and hind limb joints of each animal were collected and placed in jars containing 10% formalin for 24 h. Subsequently, the joints were placed in 10% EDTA solution for decalcification. After decalcification, the joint tissues were processed for histopathology analysis. Thin sections (5 µm) were cut from paraffin blocks, stained with H&E, and examined under a microscope. Other organs such as hearts, liver, lungs, kidneys, spleens, and adipose tissues, were also harvested, placed in jars containing 10% formalin, and processed similarly for histopathological analyses. Three H&E-stained slides of tarsal joint samples were prepared and evaluated histopathologically for each rat.

### BMD

CT scanning was conducted at Tissue Engineering Centre (UKM Medical Centre) using a Micro-CT scanner. Five rats from each group were subjected to CT-scan analysis before and after induction of CIA and at the end of the treatment period. Briefly, the rats were anaesthetised by injecting 0.2 mL of a cocktail of ketamine and xylazine and then placed in the scanning machine. The raw data obtained from the CT scanner were reconstructed using N RECON software. The reconstructed data were analysed to obtain BMD values.

### Statistical analysis

All data obtained in this study were analysed using the Statistical Package for the Social Sciences (SPSS) version 22 (SPSS Inc., Chicago, IL, USA). All data were expressed as means ± standard deviations. One-way analysis of variance followed by Bonferroni’s post-hoc test was used for comparing the experimental means between different experimental and control groups. Results with *p* values of less than 0.05 were considered statistically significant.

### Ethics approval

Ethical approval for the animal study was received from the Institutional Animal Care and Use Committee (IACUC) of the University Putra Malaysia (UPM; reference number UPM/IACUC/AUP-R042/2014).

## Supplementary information

Supplementary Figure S1.
