# Supplementary material for: Investigation of the curative effects of palm vitamin E tocotrienols on autoimmune arthritis disease in vivo
Source: Sci Rep. 2019 Nov 14;9:16793. doi: 10.1038/s41598-019-53424-7 (PMC6856359; doi:10.1038/s41598-019-53424-7)
Supplement: Supplementary file 1 — Supplementary Figure S1. [file 41598_2019_53424_MOESM1_ESM.pdf]

Investigation of the curative effects of palm vitamin E tocotrienols on autoimmune arthritis disease *in vivo*

***Zaida Zainal[1], Afiqah Abdul Rahim[1], Ammu Kutty Radhakrishnan[2], Sui Kiat Chang[3], Huzwah Khaza'ai[4]**

[1]Nutrition Unit, Department of Product Development and Advisory Services (PDAS), Malaysian Palm Oil Board, Bandar Baru Bangi, Selangor 43000, Malaysia

[2]Jeffrey Cheah School of Medicine and Health Sciences, Monash University Malaysia, Jalan Lagoon Selatan, Bandar Sunway, 47500 Subang Jaya, Selangor

[3]Department of Nutrition and Dietetics, School of Health Sciences, International Medical University, 57000 Bukit Jalil, Kuala Lumpur, Malaysia

[4]Faculty of Medicine and Health Sciences, Universiti Putra Malaysia, Serdang 43400, Malaysia

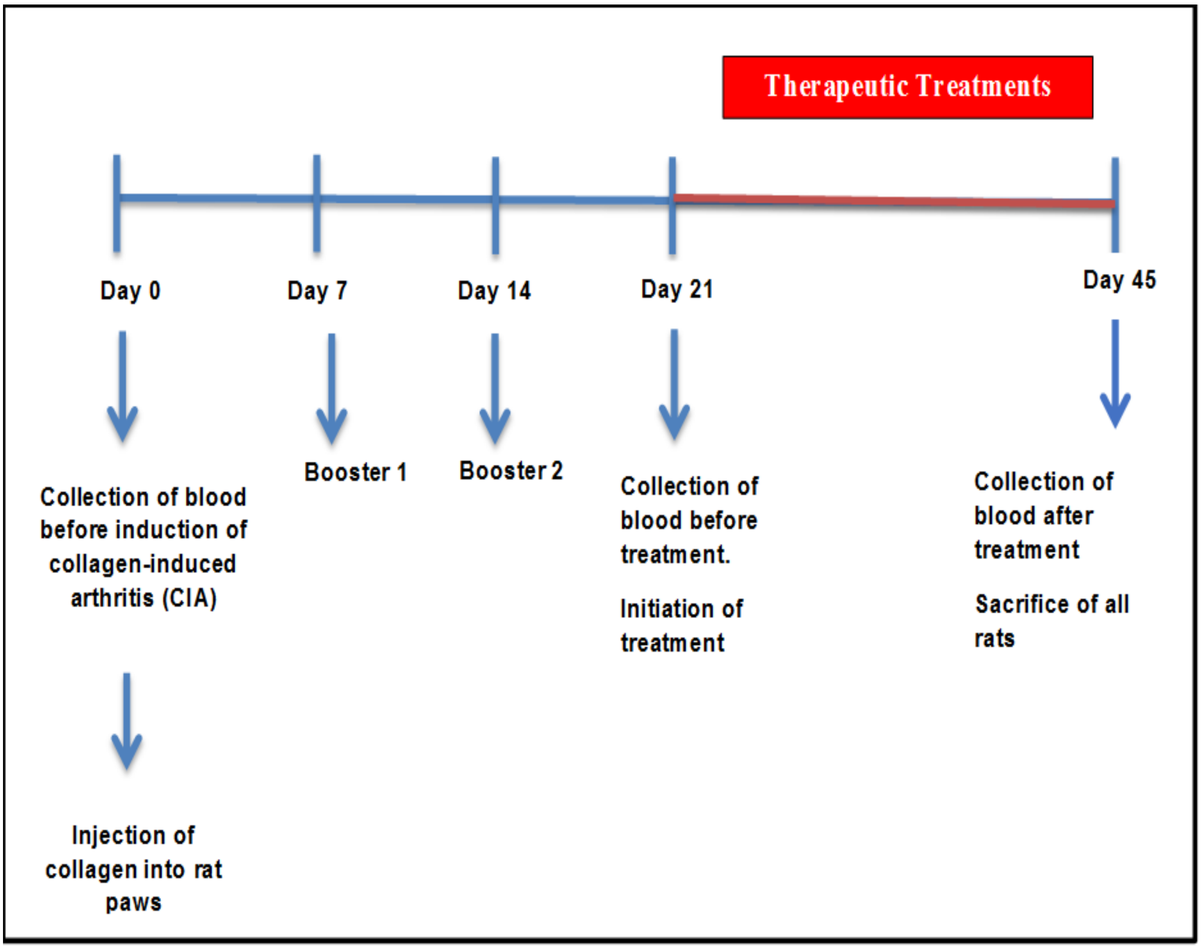

Supplementary Figure S1. Study design for DA rats induced by CIA and treated with tocotrienol. Experimental design for arthritis. Normal SPF Dark Agouti rats were injected with collagen to generate collagen-induced arthritis (CIA), except in the control group. Rats were divided into the control, arthritis, and TRF-treated groups (n = 10/group). Animals were scarified after 45 days.
